# Bidirectional traction-assisted endoscopic submucosal dissection of a sessile serrated lesion invading into the appendiceal lumen

**DOI:** 10.1055/a-2977-7641

**Published:** 2026-10-28

**Authors:** Guanyi Liu, Yunlong Cai, Yajie Wang, Bin Zhou, Long Rong

**Affiliations:** 1 Endoscopy Center 26447 Peking University First HospitalBeijingChina

A 62-year-old man was referred for endoscopic submucosal dissection (ESD) after a screening colonoscopy detected a flat lesion at the appendiceal orifice. Pre-procedural magnifying endoscopy showed the lesion growing into the appendiceal lumen, with inner border hard to identified. Narrow-band imaging and indigo-carmine staining demonstrated type II-O pit pattern ( Fig. 1 ), highly suggestive of an intraluminal-extending sessile serrated lesion (SSL). The patient and family strongly requested ESD for appendix preservation ( Video 1 ).

**Fig. 1:**
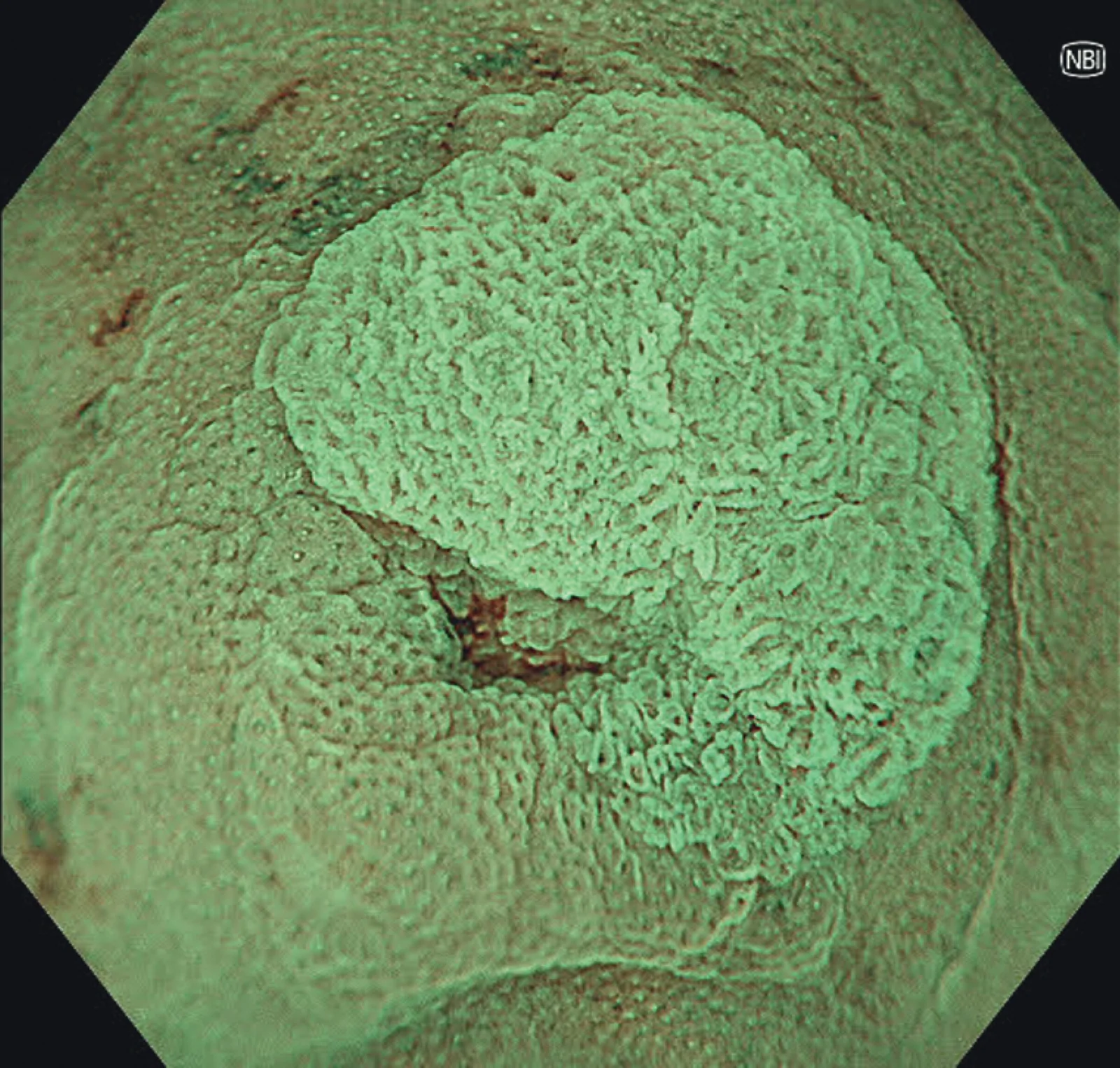
Pre-procedural magnifying endoscopy with narrow-band imaging (NBI) showed the lesion growing into the appendiceal lumen, demonstrating the type II-O pit pattern.

**Video 1** Bidirectional traction–assisted endoscopic submucosal dissection for a sessile serrated lesion invading into the appendiceal lumen. The URI for the video is as follows: .


After submucosal injection of methylene-blue-mixed normal saline, a near-circumferential mucosal incision was performed. Owing to the anatomical features of the appendiceal orifice, adequate exposure of the submucosal layer is difficult. A rubber-band-equipped clip was anchored at the right incision margin as a fulcrum, and dissection proceeded toward the appendiceal base. When the supporting effect became insufficient, a clip was used to pull the rubber band toward the left side to provide tissue exposure and outward traction of the appendiceal lumen ( Fig. 2 ). But the division of the mucosa at the appendiceal base could not be achieved. We snared and removed the left-side traction-clip using a brief electric current. A third clip grasping the rubber band provided reverse traction toward the right ( Fig. 3 ), exposing residual intraluminal tissue ( Fig. 4 ) and allowing en-bloc resection. The muscularis propria remained intact ( Fig. 5 ). Only clip-associated mucosal injury on the left was closed, and the resection site was left open. The specimen showed circumferentially normal surrounding mucosa. Postoperatively, the patient received bowel rest and prophylactic antibiotics and recovered without appendicitis. Histopathology confirmed the SSL with negative margins.

**Fig. 2:**
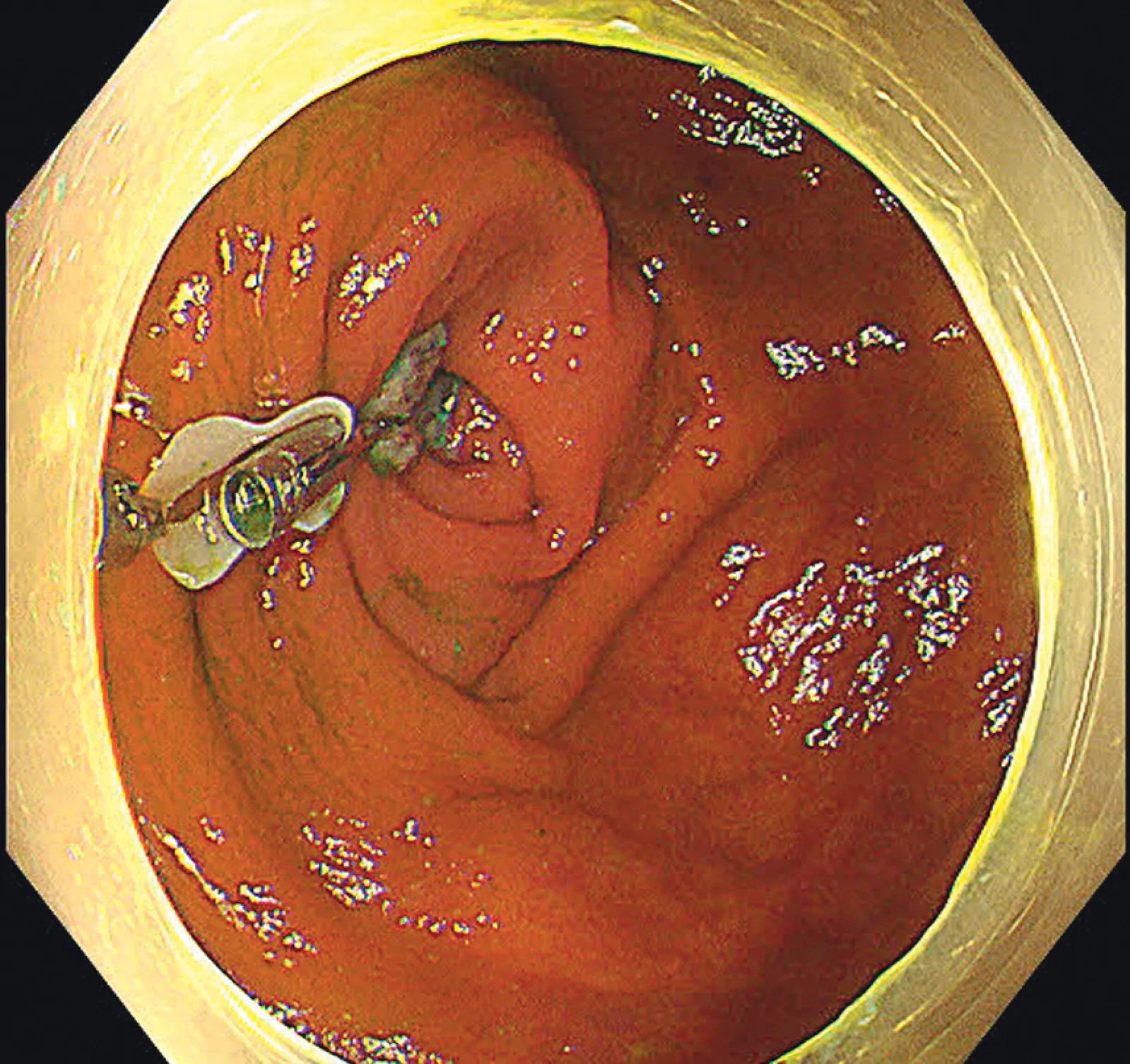
After dissection from the right margin, a clip was used to pull the rubber band toward the left side to provide tissue exposure and outward traction of the appendiceal lumen.

**Fig. 3:**
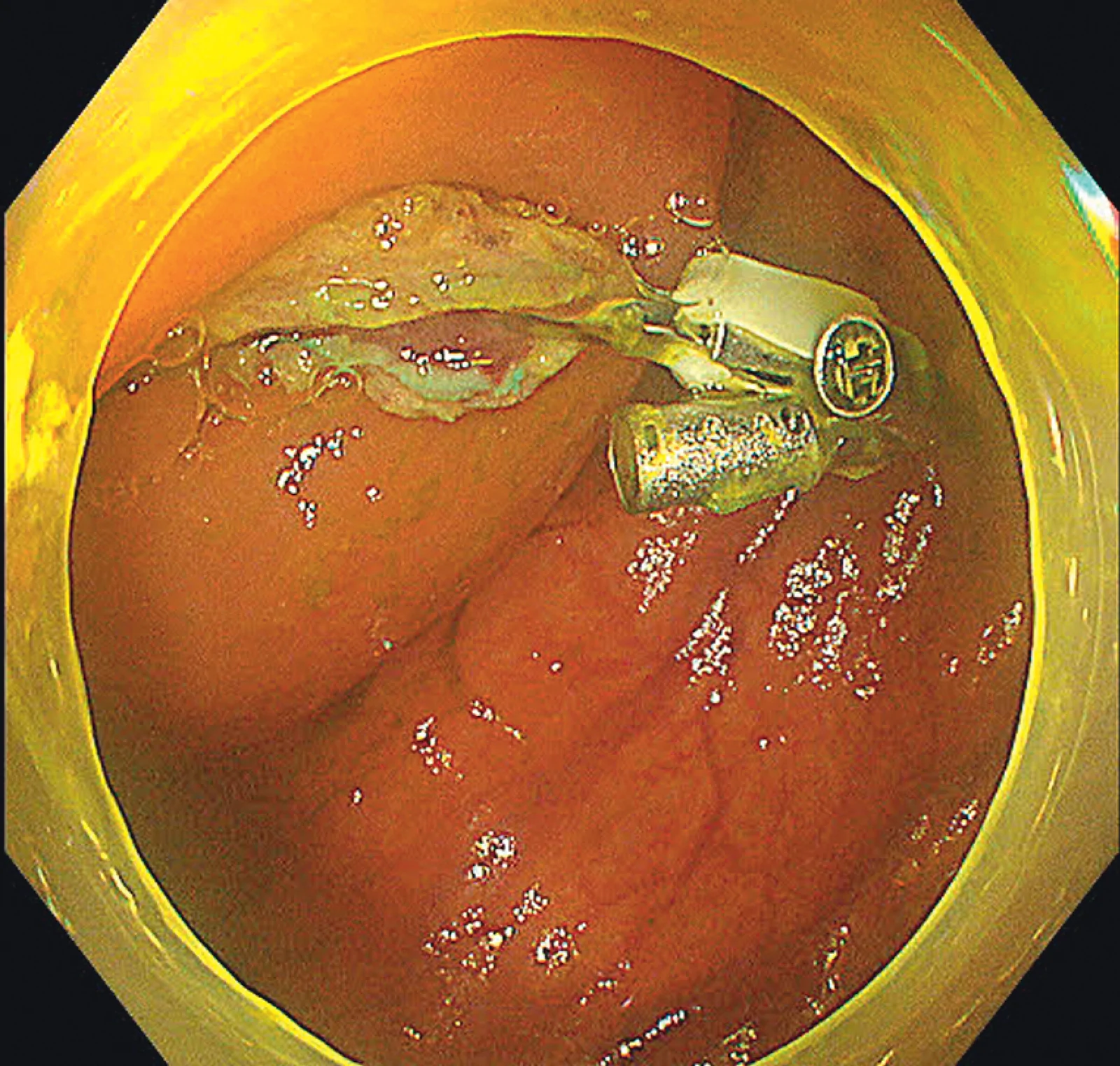
When the division of the mucosa at the appendiceal base became unachievable, we snared and removed the left-side traction-clip and use a third clip grasping the rubber band to provide reverse traction towards the right.

**Fig. 4:**
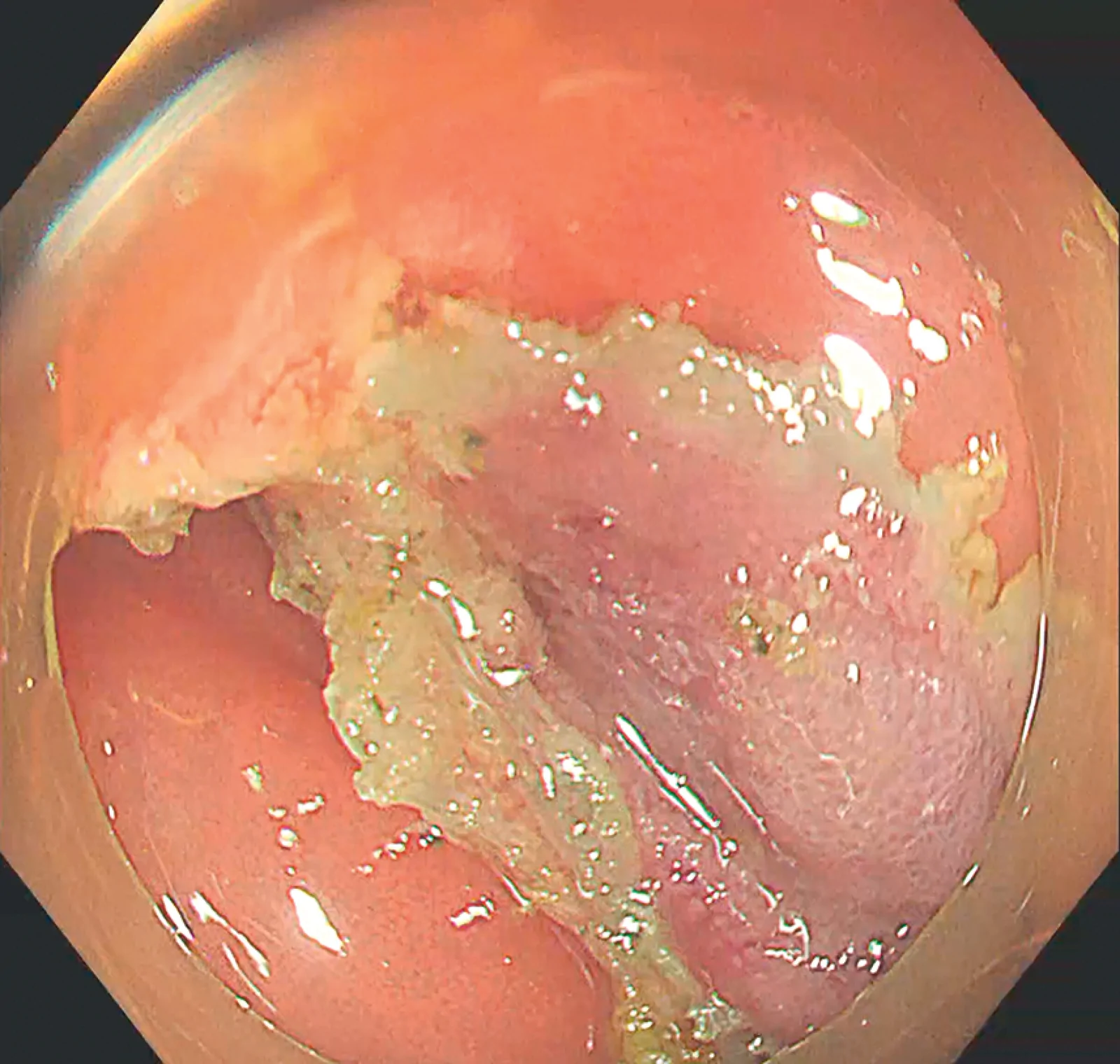
The right-side traction perfectly exposed and tensed the residual intraluminal tissue.

**Fig. 5:**
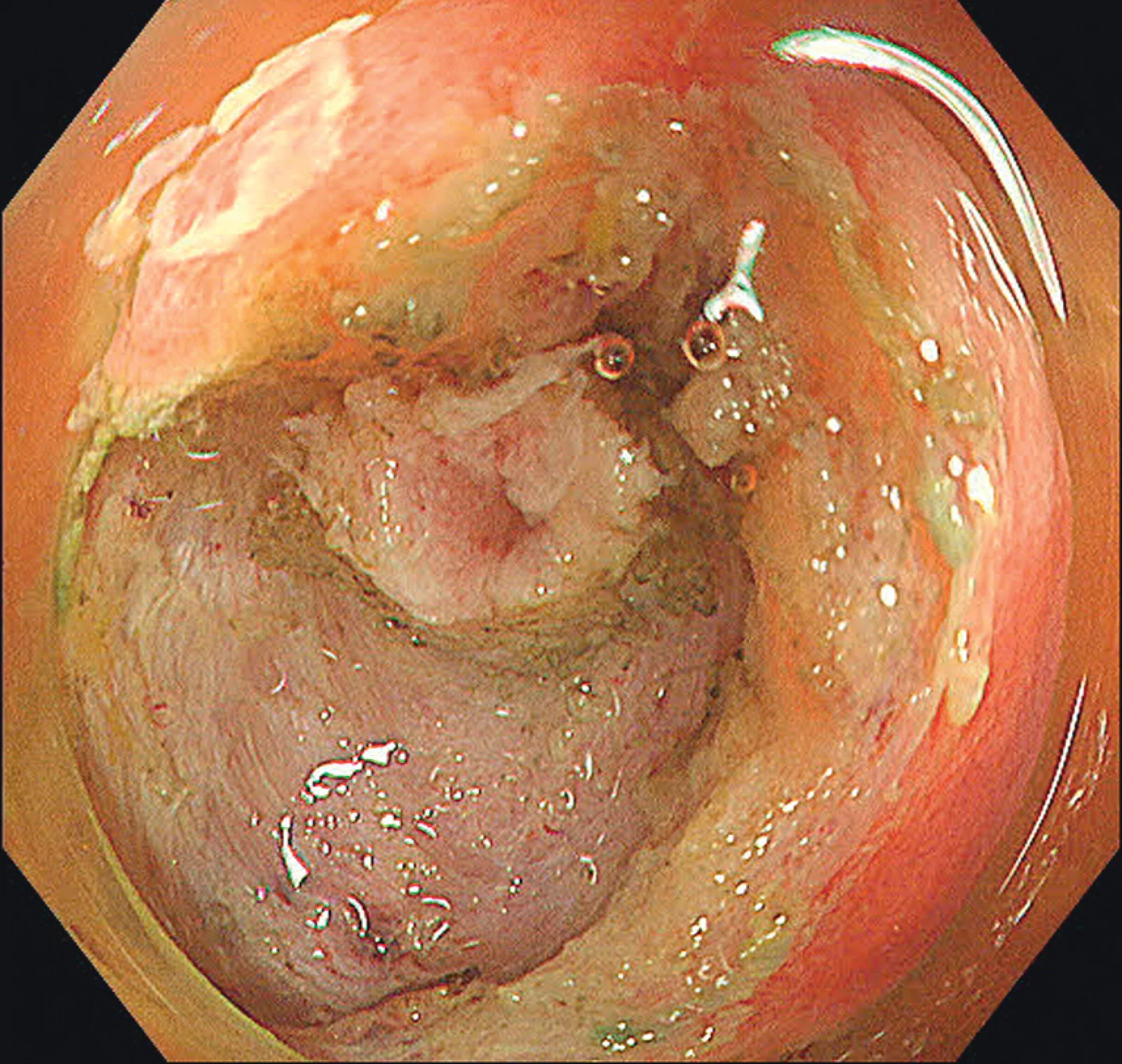
After resection, the muscularis propria of the defect remained intact and thus left open.

Appendiceal-orifice lesions frequently extend intraluminally. Conventional ESD is suitable for lesions with clear margin, while traction-assisted ESD 1​ or endoscopic appendectomy 2​ is considered for poorly demarcated lesions. Yet unidirectional conventional traction hampers deep appendiceal dissection. This case illustrates a practical bidirectional-traction technique to facilitate ESD for such lesions.

Endoscopy_UCTN_Code_TTT_1AQ_2AC

## Declaration of GenAI Use

The authors declare the use of generative artificial intelligence (AI) in the research and writing process. According to the GAIDeT taxonomy (2025), the following tasks were delegated to GAI tools under full human supervision: Adapting and adjusting emotional tone. The GAI tool used was Deepseek V4. Responsibility for the final manuscript lies entirely with the authors. GAI tools are not listed as authors and do not bear responsibility for the final outcomes.
